# Identification of Nucleic Acid Binding Sites on Translin-Associated Factor X (TRAX) Protein

**DOI:** 10.1371/journal.pone.0033035

**Published:** 2012-03-12

**Authors:** Gagan Deep Gupta, Vinay Kumar

**Affiliations:** High Pressure & Synchrotron Radiation Physics Division, Bhabha Atomic Research Centre, Mumbai, India; University of South Florida College of Medicine, United States of America

## Abstract

Translin and TRAX proteins play roles in very important cellular processes such as DNA recombination, spatial and temporal expression of mRNA, and in siRNA processing. Translin forms a homomeric nucleic acid binding complex and binds to ssDNA and RNA. However, a mutant translin construct that forms homomeric complex lacking nucleic acid binding activity is able to form fully active heteromeric translin-TRAX complex when co-expressed with TRAX. A substantial progress has been made in identifying translin sites that mediate its binding activity, while TRAX was thought not to bind DNA or RNA on its own. We here for the first time demonstrate nucleic acid binding to TRAX by crosslinking radiolabeled ssDNA to heteromeric translin-TRAX complex using UV-laser. The TRAX and translin, photochemically crosslinked with ssDNA, were individually detected on SDS-PAGE. We mutated two motifs in TRAX and translin, designated B2 and B3, to help define the nucleic acid binding sites in the TRAX sequence. The most pronounced effect was observed in the mutants of B3 motif that impaired nucleic acid binding activity of the heteromeric complexes. We suggest that both translin and TRAX are binding competent and contribute to the nucleic acid binding activity.

## Introduction

TRAX protein was initially identified in a yeast two hybrid screen as a protein that interacts with translin, a ssDNA and RNA binding protein [1]–[3]. Translin and TRAX proteins share homology and have been evolutionarily conserved in eukaryotes [4]–[6]. Evidence that these endogenous proteins interact *in vivo* has been provided by immuno- precipitation studies [7], [8]. Furthermore, deletion of translin in mice leads to complete loss of TRAX protein, without affecting TRAX mRNA levels, indicating that the stability of TRAX protein is dependent on its interaction with translin [9], [10]. Similar results have been reported in *Drosophila melanogaster* (drosophila) and *Schizosaccharomyces pombe* (*S. pombe*) demonstrating that the close interaction between translin and TRAX has been conserved over the course of evolution [11], [12].

As the translin-TRAX complex binds to both ssDNA and RNA, it has been implicated in a variety of cellular functions. Initially, it was thought that its ability to bind to ssDNA indicated its involvement in DNA repair [3], [13]. This view has been supported by the observation that DNA damaging agents induce TRAX's interaction with the nuclear C1D as a part of a cellular DNA repair response [14]. As the complex also binds to RNA *in vitro*, two recent studies have focused attention on its possible role in regulating RNA trafficking and processing. One provides evidence that the complex mediates dendritic trafficking of *BDNF* mRNA, a transcript that plays a critical role in synaptic plasticity processes [15]. The other suggests that the translin-TRAX complex functions as a key activator of siRNA-mediated silencing in drosophila [16]. In addition, TRAX has been found to suppress proliferation of PC12 cells in the presence of p53 blockade [17] and to regulate mRNA levels encoding growth-associated protein (GAP)-43 in retinal ganglion cells [18]. Also TRAX interacts with mammalian phospholipase Cβ1 [19].

To understand how the translin-TRAX complex exerts its cellular effects, it would be helpful to define how it binds to ssDNA or RNA. Initial characterization of translin and TRAX revealed that translin, but not TRAX alone, is able to form a homomeric complex capable of binding ssDNA or RNA. Analysis of the human, mouse or yeast translin homomeric complexes demonstrated that they form octameric complexes, while chicken translin forms a decameric complex [20]. Furthermore, substantial progress has been made in identifying nucleic acid binding sites in translin homomeric complexes. For example, mutations of residues in one of the basic domains, referred to as basic-2 (translinB2 here), markedly reduces the ssDNA or RNA binding activity of the translin homomeric complex [20]. However, translin constructs with mutations in the B2 region are able to form heteromeric complexes with TRAX that are fully active in gel-shift binding assays [6], [21]. Also, heteromeric complex between wild-type translin and TRAX proteins showed stronger binding to DNA than RNA [21], [22]. The TRAX protein possesses nuclear localization signal and in the nucleus could change its protein partners between translin and C1D [22]. These observations hint whether physiological effects of the TRAX are dependent on its nucleic acid binding activity, though TRAX on its own is believed not bind to RNA or DNA [6], [21], [22]

We have conducted studies aimed at deducing nucleic acid binding characteristics of TRAX and in defining the binding sites. Our results show that TRAX binds to ssDNA and two regions in TRAX, designated TRAXB2 and TRAXB3, mediate nucleic acid binding activity of the heteromeric complex. We also found that translin B3 motif, corresponding to TRAX B3 residues, contributes towards its DNA binding activity.

## Materials and Methods

### Materials

dNTPs and T4 DNA ligase were obtained from Roche (Germany), and restriction enzymes and T4 polynucleotide kinase from New England BioLabs (Hitchin, UK). QuickChange site-directed mutagenesis kit and *PfuTurbo*DNA polymerase were obtained from Stratagene (La Jolla, USA). Plasmid *p*QE9 was obtained from Qiagen (QIAGEN India Pvt. Ltd., India), *E. coli* BL21(DE3) cells [*E. coli* B F^−^ *dcmompThsdS*(r_B_^−^, m_B_^−^) *galλ*(DE3)], and plasmids *p*ET21a and *p*ET28a were obtained from Novagen (Madison, USA). Chromatography media were obtained from Amersham-Pharmacia (Uppsala, Sweden). Oligonucleotides for cloning, ssDNA of Bcl-CL1 sequence, [α-^32^P]GTP and [γ-^32^P]ATP were synthesized at BRIT (Navi Mumbai, India). Other fine chemicals were procured from SRL (Mumbai, India).

### Prediction of DNA-binding residues

The DNA-binding sites in human TRAX and translin sequences were predicted using the DP-Bind web-server [23; http://lcg.rit.albany.edu/dp–bind/]. The analysis was performed with PSSM-based encoding which uses a position-specific scoring matrix generated by PSI-BLAST [24]. The DNA-binding residues were identified from the consensus that retained only high confidence predictions and were categorized in five motifs (B1–5) on TRAX and translin sequences.

### Recombinant plasmids

The *p*QE9-*translin* and *p*ET28a-*trx* constructs, used to express human translin and human TRAX, were those used earlier [25]. The constructs were kind gifts from Professors M. Kasai, National Institute of Health, Tokyo, Japan and N.B. Hecht, University of Pennsylvania, USA, respectively. Mutants of the putative nucleic acid binding motifs B2 and B3 on the TRAX sequence, (TRAXB2- ^115^QFHRA^119^ to ^115^LFNAA^119^) (*p*ET28a-*trxB2* construct) and (TRAXB3- ^241^KKLY^244^ to ^241^NTLN^244^) (*p*ET28a-*trxB3* construct), were synthesized by QuickChange site-directed mutagenesis kit using *p*ET28a-*trx* as template and complementary primers pairs (Table S1). The constructs were used to express TRAX variants with poly-histidine (6×His) tag at their N-termini. Mutants of the B2 and B3 nucleic acid binding motifs on the translin sequence, (translinB2; ^86^RFHEH^90^ to ^86^TFNEN^90^) (*p*QE9-*translinB2* construct) and (translinB3; ^192^RKRY^195^ to ^192^TNSN^195^) (*p*QE9-*translinB3* construct), were synthesized by the PCR overlap extension method using the overlapping primer pairs (Table S1). The constructs *p*ET21a-*translin*, *p*ET21a-*translinB2* and *p*ET21a-*translinB3*, were also synthesized by cloning mutant and wild-type *translin* genes into the *Nde*I and *Bam*HI sites of *p*ET21a plasmid to express translin proteins without any tag, to be used for co-expression system. The nucleotide sequences of all the cloned genes were confirmed by DNA sequencing using an ABI automated DNA sequencer. A summary of all the constructs used for testing DNA binding activity is listed in Table 1.

**Table 1:** Details of the constructs and expression systems used, and ssDNA binding characteristics of the purified proteins.

Protein/Complex (abbreviation)	Expression system	Activity^1^
Human translin (translin)	*p*QE9-*translin* transformed into *E. coli* BL21 (DE3)	+ + +
Mutant of basic-2 motif of human translin (translinB2)	*p*QE9-*translinB2* transformed into *E. coli* BL21 (DE3)	ND^2^
Mutant of basic-3 motif of human translin (translinB3)	*p*QE9-*translinB3* transformed into *E. coli* BL21 (DE3)	ND
Complex of human translin and TRAX proteins (translin-TRAX)	*p*ET21a-*translin* and *p*ET28a-*trx* co-transformed into *E. coli* BL21(DE3)	+ + + +
Complex of human translin with human B2 mutant TRAX (translin-TRAXB2)	*p*ET21a-*translin* and *p*ET28a-*trxB2* co-transformed into *E. coli* BL21(DE3)	+ + + +
Complex of human translin with human B3 mutant TRAX (translin-TRAXB3)	*p*ET21a-*translin* and *p*ET28a-*trxB3* co-transformed into *E. coli* BL21(DE3)	+
Complex of human B2 mutant translin with human TRAX (translinB2-TRAX)	*p*ET21a-*translinB2* and *p*ET28a-*trx* co-transformed into *E. coli* BL21(DE3)	+ + + +
Complex of human B2 mutant Translin with human B2 mutant TRAX (translinB2-TRAXB2)	*p*ET21a-*translinB2* and *p*ET28a-*trxB2* co-transformed into *E. coli* BL21(DE3)	+ + +
Complex of human B2 mutant translin with human B3 mutant TRAX (translinB2-TRAXB3)	*p*ET21a-*translinB2* and *p*ET28a-*trxB3* co-transformed into *E. coli* BL21(DE3)	ND^2^
Complex of human B3 mutant translin with human TRAX (translinB3-TRAX)	*p*ET21a-*translinB3* and *p*ET28a-*trx* co-transformed into *E. coli* BL21(DE3)	ND
Complex of human B3 mutant Translin with human B2 mutant TRAX (translinB3-TRAXB2)	*p*ET21a-*translinB3* and *p*ET28a-*trxB2* co-transformed into *E. coli* BL21(DE3)	ND
Complex of human B3 mutant translin with human B3 mutant TRAX (translinB3-TRAXB3)	*p*ET21a-*translinB3* and *p*ET28a-*trxB3* co-transformed into *E. coli* BL21(DE3)	ND

ND-Activity was not detected under experimental conditions using radiolabeled ssDNA.

1 The relative DNA-binding activity represented as four bins of 25% each.

2 Weak activity detected with higher concentrations of proteins and DNA.

### Expression and purification of the proteins

The *E. coli* BL21(DE3) cells harboring *p*ET21a-*translin* were co-transformed with *p*ET28a-*trx*, *p*ET28a-*trxB2* or *p*ET28a-*trxB3* constructs to express the complexes of wild-type human translin with wild-type or mutant TRAX proteins. Similarly, the complexes of human mutant translin (translinB2 or translinB3) with TRAX (wild type or mutants) were expressed by co-transforming the *p*ET28a-*trx*, *p*ET28a-*trxB2* or *p*ET28a-*trxB3* into the *E. coli* BL21 (DE3) cells harboring *p*ET21a-*translinB2* or *p*ET21a-*translinB2* construct. These all 9 complexes were expressed as 6×His-TRAX complexed with untagged translin. The co-transformants were selected and maintained using two antibiotics, based on the resistances conferred by the transforming vectors. The homomeric translin proteins (wild-type translin, translinB2 mutant or translinB3 mutant) were expressed with N-terminal 6×His tag in *E. coli* BL21(DE3) cells harboring *p*QE9 constructs (*p*QE9-*translin*, *p*QE9-*translinB2* or *p*QE9-*translinB3*). The expression of translin and TRAX proteins was induced by isopropyl-β-D-1-thio galactopyranoside (IPTG). Translin proteins or translin-TRAX complexes (Table 1) were purified using chelating-sepharose FF column (Nickel(II)-iminodiacetic acid; Ni-IDA) followed by anion-exchange high Q-sepharose column chromatography. The purified proteins were incubated with DNase1 and RNaseA overnight at 20°C followed by purification using Ni-IDA column. The purification protocol for heteromeric complexes ensured recovery of only the translin-TRAX complexes from the affinity matrix because translin was untagged and 6×His tagged TRAX was insoluble when expressed alone.

### Gel filtration and Circular dichroism analysis

The purified translin proteins and translin-TRAX complexes were loaded on the Superdex™ 200 10/300 GL column (GE Healthcare) for final purification step as well as for molecular weight determination. The Superdex™ 200 column was calibrated with gel filtration molecular weight markers (Amersham-Pharmacia; Carbonic anhydrase, 29 kDa; Alcohol dehydrogenase, 150 kDa; β-amylase, 200 kDa; Ferritin, 440 kDa). The eluted peak of each independent gel filtration experiment was adjudged on SDS-PAGE. The purified proteins were concentrated to 5–7 mg mL^−1^. The protein concentrations were estimated by modified Lowry's method [26] using bovine serum albumin (BSA) as the standard.

The far-UV circular dichroism (CD) spectra for the purified translin proteins (0.3 mg mL^−1^) and translin-TRAX complexes (0.3 mg mL^−1^) were recorded in the wavelength range of 200–260 nm at 25°C with JASCO spectro-polarimeter (model J-810). Each spectrum was averaged for three scans. The observed spectra were deconvoluted into their secondary structure content using K2D2 software [27].

### DNA-binding assay

Electrophoresis mobility shift assays (EMSA) were performed to assess DNA binding-activity of the DNase1 and RNaseA treated proteins/complexes using Bcl-CL1 ssDNA (GCCCTCCTGCCCTCCTTCCGCGGG) as probe. The molecular masses of the heteromeric complexes used were ∼300 kDa, whereas oligomers of human translin were 236 kDa, respectively. The probe was 5′ end-labeled using T4 polynucleotide kinase (3 U reaction^−1^) and [γ-^32^P]ATP in a 50 µL reaction buffer at 37°C for 60 minute followed by heat inactivation of the enzyme at 65°C for 30 minutes. The radiolabeled probe was purified by spin column MicroSpin™ G-25 (GE Healthcare). Translin (50 nM of octameric translin) and translin-TRAX complexes (100 nM of 300 kDa translin-TRAX complex) were incubated with the labeled-DNA probe (100 nM) in binding buffer B1 (25 mM Tris-HCl pH 8.0, 100 mM NaCl, 1 mM EDTA) for 30 minute. The 10 µL of reaction mixtures were separated by electrophoresis on a 4.5% native-PAGE in 0.5× Tris-borate-EDTA (TBE) buffer. The dried gels were exposed to X-ray film overnight. The intensity of the shifted DNA-protein complexes was estimated using a gel documentation system from Syngene (Synoptics, Cambridge, England).

The identity of proteins in gel-shifted bands from the EMSA analysis performed with 100-fold higher concentrations of DNA and proteins was adjudged by SDS-PAGE analysis. The DNA in this experiment was stained with ethidium bromide and varying concentrations of translin proteins (1 and 5 µM) and translin-TRAX complexes (2 and 10 µM) were incubated with 10 µM of the 24-mer unlabeled-DNA oligo in 20 µL reaction volume for 30 minutes. The products were resolved by electrophoresis on 1.5% agarose gel and stained with ethidium bromide. The agarose regions containing gel-shifted bands were excised and adjudged on 12% SDS-PAGE, which confirmed that both translin and TRAX proteins were components of translin-TRAX∶DNA complexes.

### Photochemical crosslinking and purification of TRAX-DNA covalent complex

To detect the direct binding of ssDNA with TRAX, the radiolabeled DNA probe was crosslinked with translin-TRAX complex using KrF excimer UV laser (COMPexPro 205) at 248 nm. Translin (50 pmol of octameric translin) and translin-TRAX complexes (50 pmol of the 300 kDa heteromeric complex) were individually incubated with 50 pmol of labeled-DNA probe for 30 min in 50 µL binding buffer (25 mM Tris-HCl pH 8.0, 100 mM NaCl, 1 mM EDTA). Each of the protein∶DNA mixtures were irradiated in a quartz cell (Eppendorf UVette) for 1 minute at 1-pulse/sec imparting about 2400 mJ of total energy. BSA∶DNA mixture was also irradiated under identical conditions as a negative control. To isolate TRAX-DNA crosslinked complex, the irradiated heteromeric complex was disrupted in the presence of 8 M urea followed by 5 minutes incubation in boiling water bath. The reaction mixture was loaded onto Ni-IDA spin-column pre-equilibrated with binding buffer (25 mM Tris-HCl, pH 8.0, 8 M urea, 50 mM imidazole and 100 mM NaCl). The matrix beads were extensively washed with binding buffer. The bound protein was eluted with elution buffer (25 mM Tris-HCl, pH 8.0, 8 M urea, 1 M imidazole and 100 mM NaCl). This protocol resulted in purification of denatured TRAX and its covalent complexes as the 6×His tag was fused only with TRAX sequence. The purification of TRAX alone was further confirmed by mass spectrometry of the purified protein component obtained by disrupting pure translin-TRAX complex in an independent experiment. The TRAX-DNA complex along with irradiated translin-DNA complex and pre-stained molecular weight markers were resolved on 12% SDS-PAGE. The dried gel was autoradiographed on X-ray Film. The positions of TRAX-DNA and translin-DNA complexes on autoradiograph of the SDS-PAGE were estimated from the mobility of the pre-stained marker proteins. To adjudge the extent of protein-protein crosslinking, purified translin was mixed with [α-^32^P]GTP and the mixture was irradiated for 2400 mJ laser energy under conditions identical to that for protein-DNA complexes.

## Results

### Prediction of DNA-binding residues on TRAX

**Figure 1:**
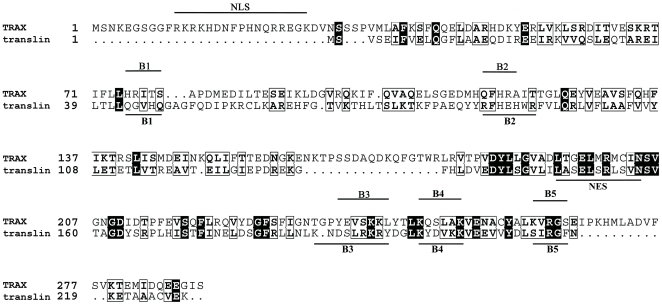
Alignment of TRAX and translin sequences. The amino acid sequences of the TRAX and translin proteins of human and drosophila (UniProtKB/Swiss-Prot Q99598, Q8INE1, Q15631 and Q7JVK6, respectively) were aligned by ClustalW [33]. For clarity, only sequences of human TRAX (TRAX) and human translin (translin) are shown. The identical amino acid residues are shaded and similar residues are boxed. The five putative DNA binding regions (B1–5) predicted by the DP-Bind server and the nuclear localization signal (NLS) on the TRAX sequence are identified. Also marked are five basic regions (B1–5) on translin sequence and its nuclear export signal (NES). The figure was prepared with ESPript [34].

**Figure 2:**
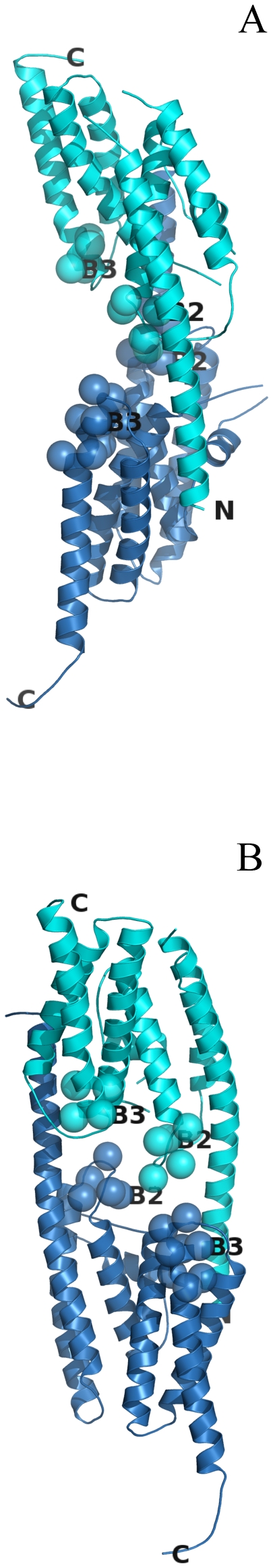
Cartoon showing close proximity of B2 and B3 motifs on human translin-TRAX heterodimer. Different views (**A**,**B**) of cartoon showing B2 and B3 motifs (spheres) of translin (cyan) and TRAX (blue) in translin-TRAX heterodimer. The figure was prepared using atomic coordinates of translin-TRAX complex structure (PDB code, 3PJA) and PyMol suite [35].

Candidate DNA-binding residues in the human TRAX protein were identified based on the consensus sites detected by the DP-Bind web server. This algorithm predicted most of the 22 N-terminus amino acids as DNA-binding residues, as well as five additional DNA binding motifs (B1, ^75^HRITS^79^; B2, ^115^QFHRA^119^; B3, ^237^YEVSKKL^243^; B4, ^247^KQSLAK^252^ and B5, ^261^KVRGS^265^). The five motifs (B1–5) were also predicted to be present in human translin sequence (Fig. 1). The previous studies had demonstrated that substitutions in B2 and B3 motifs in translin, identified here, resulted in loss of ssDNA binding activity [21], [28]. The B2/B3 motifs of TRAX are disposed similar to those of translin monomers in the structure of translin-TRAX heterodimer (Fig. 2). Accordingly, to test the role of the B2 and B3 motifs of TRAX in nucleic acid binding activity of the heteromeric translin-TRAX complex, we generated TRAX constructs in which amino acid residues of either the B2 or B3 motifs were mutated as follows: TRAXB2 - ^115^QFHRA^119^ to ^115^LFNAA^119^; TRAXB3 - ^241^KKLY^244^ to ^241^NTLN^244^.

### Oligomeric status and structural content of translin-TRAX complexes

**Figure 3:**
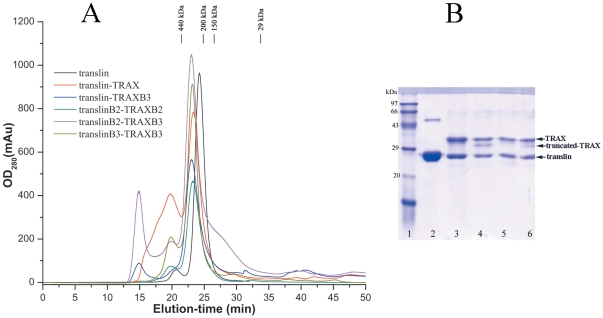
Gel filtration analyses of human translin and translin-TRAX complexes. **A**) A profile overlay of Superdex 200 gel-filtration chromatography of human translin and translin-TRAX complexes. The major peaks corresponding to molecular masses of 295 kDa for the complexes and 236 kDa for the translin were used for DNA-binding assays. **B**) The translin/TRAX protein complexes purified by gel-filtration chromatography were adjudged on SDS-PAGE. Molecular weight markers, lane1; human translin, lane 2; translinB2-TRAXB3 complex, lane 3; translin-TRAXB3 complex, lane 4; translinB2-TRAXB2 complex, lane 5 and wild-type translin-TRAX complex, lane 6. Nearly equimolar stochiometry of translin and TRAX proteins in the heteromeric complexes could be estimated from the band intensities. Truncation in TRAX protein was observed on storage. The truncation site at the C-terminus was confirmed by N-terminal sequencing and MALDI-TOF analyses. Integrated intensities of both the TRAX bands were summed to estimate translin/TRAX stoichiometry.

Prior to testing the impact of these mutations in TRAX on the nucleic acid binding activity of the translin-TRAX complex, we checked whether they altered its size or composition. The molecular masses of the heteromeric complexes formed by translin variants with either wild type TRAX or TRAXB2 or TRAXB3 proteins were determined to be ∼300 kDa by gel-filtration chromatography (Fig. 3A). The eluted proteins were adjudged on 12% SDS-PAGE. The intensities of translin and TRAX bands showed nearly equimolar ratio of the two proteins in the heteromeric complexes (Fig. 3B). By comparison, the molecular mass of homomeric translin complexes generated by either wild-type translin or translin constructs containing mutations in B2 or B3 domains (^86^RFHEH^90^ to ^86^TFNEN^90^; ^192^RKRY^195^ to ^192^TNSN^195^) were determined to be approximately 236 kDa, consistent with earlier reports that mutations in the basic-2 (B2) motif do not affect the octameric status of human translin [20].

**Figure 4:**
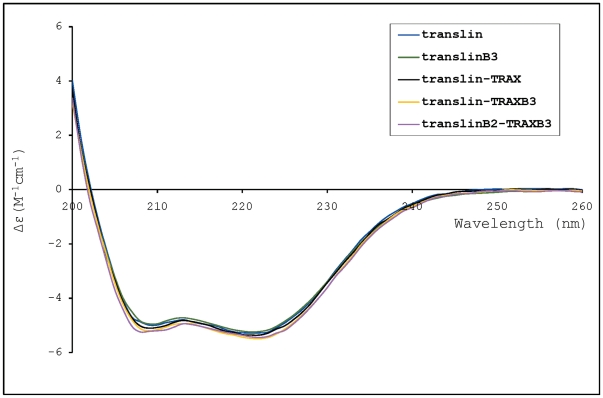
Circular dichroism (CD) analysis of the proteins in the wavelength range of 200–260 nm. The characteristic negative bands of α-helical proteins at 222 nm and 208 nm are observed for all the proteins and complexes. The CD spectrum of human translin essentially overlaps with those of translin-TRAX complexes. For clarity, CD spectra is shown for a few of the protein/complexes. The CD spectra presented are of human translin (translin), human mutant translinB3 (translinB3), human translin-TRAX complex (translin-TRAX), and complexes of mutants of human TRAXB3 and translin (translin-TRAXB3 and translinB2-TRAXB3).

We compared the CD spectra of homomeric translin and heteromeric translin-TRAX complexes and found excellent agreement between CD spectra of heteromeric and homomeric complexes in the wavelength range of 200–260 nm with the known crystal structures of translin and translin-TRAX complexes (Fig. 4). The observed characteristic negative bands of α-helical proteins at 222 nm and 208 nm are in conformity with all-helical structure of translin and TRAX proteins. The CD analysis also suggests that mutations in the nucleic acid binding motifs of translin and TRAX do not alter the secondary structural content of the complexes.

### DNA-binding activity

**Figure 5:**
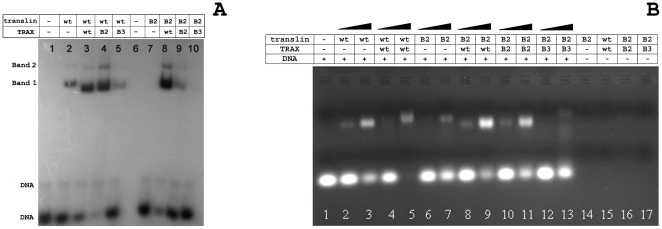
DNA-binding activity of the proteins/complexes. **A**) [γ-^32^P]-labeled Bcl-CL1 24-mer ssDNA (100 nM) was incubated with human translin (50 nM of octameric translin) and translin-TRAX complexes (100 nM of octameric translin-TRAX complex). The mixtures were resolved on the 4.5% native-PAGE in TBE buffer. Prior to EMSA analysis all the purified samples were treated with DNase1 and RNaseA overnight at 20°C followed by purification using Ni-IDA chelating sepharose column. Lane 1, only DNA; lane 2, human translin; lane 3, translin-TRAX complex; lanes 4 and 5, complexes of human translin with the B2 and B3 mutants of human TRAX (translin-TRAXB2 and translin-TRAXB3), respectively; lane 6, Blank; lane 7, basic-2 region mutant of human translin (translinB2); lanes 8, 9 and 10, complexes of translinB2 with human TRAX, TRAXB2 and TRAXB3 (translinB2-TRAX, translinB2-TRAXB2 and translinB2-TRAXB3), respectively. Two bands with retarded mobility, compared to DNA alone, were observed in active protein complexes. Band 1 presumably corresponded to octameric complex while band 2 with very weak intensity belonged to higher oilgomers. **B**) Unlabled Bcl-CL1 24-mer ssDNA (10 µM) was incubated with human translin (1 µM and 5 µM, lanes 2 and lane 3 respectively), wild-type translin-TRAX complex (2 µM and 10 µM, lanes 4 and 5 respectively), translinB2 mutant (1 µM and 5 µM, lanes 6 and 7 respectively), translinB2-TRAX (2 µM and 10 µM, lanes 8 and 9 respectively), translinB2-TRAXB2 (2 µM and 10 µM, lanes 10 and 11 respectively) and translinB2-TRAXB3 (2 µM and 10 µM, lanes 12 and 13 respectively), and the reaction mixtures were resolved on 1.5% agarose gel were stained with ethidium bromide. At higher concentrations the ssDNA binding activity of translinB2-TRAXB3 complex was weakly detectable with ethidium bromide. This was not observed in autoradiograms with low concentrations of radiolabeled DNA and the protein complex.

The DNA-binding activities of homomeric translin and heteromeric translin-TRAX complexes were monitored by electrophoresis mobility shift assays using the Bcl-CL1 probe, which is a 24-mer ssDNA oligo used in initial studies characterizing the nucleic acid binding properties of translin and TRAX [3]. Two bands with retarded migration (bands 1 and 2) were observed for the active protein complexes, compared to DNA alone (lane 1, Fig. 5A). However, intensity of the slow migrating band 2 was very weak, compared to band 1. Band 2 thus corresponded to higher oligomer observed in low-abundance in gel-filtration and native-PAGE analyses (Fig. S1) of the pure proteins. Similar band pattern was also observed in earlier studies aimed to deduce the nucleic acid binding properties of translin or translin-TRAX complex proteins [20], [21]. The heteromeric translin-TRAX complex displayed enhanced ssDNA binding activity compared to the homomeric translin complex (lanes 3 and 2; Fig. 5A). To test the predicted role of the B2 and B3 motifs of TRAX in nucleic acid binding activity, we examined the ssDNA binding activities of the heteromeric complexes formed by wild type translin with the TRAXB2 or TRAXB3 mutant constructs (Table 1). While the binding activity displayed by the translin-TRAXB2 complex is comparable to that of the wild type translin-TRAX complex (lane 4), the binding activity of the translin-TRAXB3 complex is markedly reduced (lane 5, Fig. 5A).

We further tested the effect of substitutions in B2/B3 motifs of TRAX in DNA binding ability of the heteromeric complex constructed using translinB2 or translinB3 mutants. Interestingly, both wild type TRAX and TRAXB2 mutants could rescue activity of translin B2 mutation (Fig. 5A, lanes 8 and 9; Fig. 5B, lanes 9 and 11). However, heteromeric complex of translinB2-TRAXB3 were observed to be fully inactive in the study with radiolabeled ssDNA probe (lane 10, Fig. 5A). Consistent with previous studies, homomeric mutant translinB2 did not show any DNA binding activity when used in low concentration with the radiolabeled probe (lane 7, Fig. 5A). Also, translinB3 mutant did not show detectable DNA binding activity (Fig. S2). Similar results were reported for the *S. pombe* translin where substitution in R210 and R211 (equivalent to R192 and K193 of human translinB3 motif) dramatically reduced DNA-binding activity [28]. Surprisingly, neither of the wild-type translin or TRAX proteins could rescue activity of TRAXB3 or translinB3 mutants (Fig. S2). To detect weak binding affinities, the assays were also performed with high concentrations of unlabeled DNA (10 µM) and proteins (typically, 1 and 5 µM of octameric translin proteins, and 2 and 10 µM of octamer of heteromeric complex). Although the band intensities showed trend of binding affinities similar to that observed with labeled DNA, however, weak binding was detected even with the heteromeric translinB2-TRAXB3 and homomeric translinB2 mutants (Fig. 5B). Protein/complexes alone were also loaded to confirm that detected intensity is not due to artifact.

Since very similar elution profile on gel-filtration column and nearly identical CD-spectra were observed for all the complexes, the data suggests that alterations in the DNA binding activities can be attributed to the substitution in DNA binding motifs. Taken together it can be concluded both B2 and B3 motifs of TRAX contribute to DNA binding activity in the context of the heteromeric complex, with mutations of the B3 site having clearly more pronounced effect.

### Photochemical crosslinking of ssDNA with TRAX

**Figure 6:**
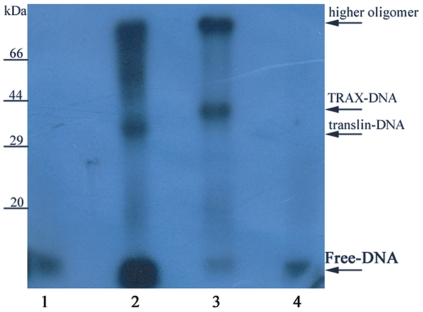
Autoradiograph showing protein-DNA crosslinking. Purified translin, translin-TRAX complex and BSA were mixed with [γ-^32^P]-labeled Bcl-CL1 24-mer ssDNA. The mixtures were irradiated with UV-laser imparting total of 2400 mJ energy of 248 nm wavelength. Post-irradiation, the translin-TRAX complex was disrupted and TRAX was purified using affinity tag. The protein∶DNA complexes (translin-DNA, lane 2; TRAX-DNA, lane 3, BSA∶DNA, lane 4) and DNA alone (lane 1) were resolved on 12% SDS-PAGE and dried gel was autoradiographed on X-ray film. The translin-DNA and TRAX-DNA complexes differ by expected 6 kDa in molecular mass. The BSA, used as a negative control, did not show presence of protein-DNA covalent complex in the post-irradiated mixture. The intensities of free DNA band in lane 1 and 4 are much weaker, as only 5 µL of the irradiated BSA∶DNA mixture was loaded, compared to 20 µL of the translin∶DNA mixtures.

The radiolabeled ssDNA probe was crosslinked with homomeric translin and heteromeric translin-TRAX complex using UV-laser. The BSA∶DNA mixture was used as a negative control in the crosslinking experiment. For unambiguous detection of TRAX-DNA covalent complex, the irradiated heteromeric translin-TRAX complex was disrupted by boiling with 8 M urea. TRAX/TRAX-DNA complex was purified with Ni-IDA matrix spin-column using 6×His tag in TRAX. The protocol resulted in purification of TRAX or its covalent complexes, as verified by mass spectrometry. The eluent of the disrupted heteromeric complex containing TRAX and TRAX covalent complexes, and irradiated translin∶DNA mixture were resolved on 12% SDS-PAGE. The translin-DNA and TRAX-DNA complexes were clearly detected on autoradiogram. The TRAX-DNA complex (lane 3, Fig. 6), migrates close to the 44 kDa marker protein and thus corresponds to TRAX molecule (35.3 kDa) covalently linked with one ssDNA moiety (∼7.6 kDa). As expected, yield of the covalent complex was very poor. A band of very weak intensity (relative to the unliganded protein) was observed on silver-stained gel SDS-PAGE (Fig. S3). The migration of this band corresponded to difference in the molecular masses between nucleic acid complexed and uncomplexed TRAX protein. The UV-crosslinked heteromeric complex with 43-mer ssDNA probe in an independent experiment showed expected shift of ∼6 kDa in the TRAX-DNA band (Fig. S3). As compared to TRAX-DNA covalent complex observed in autoradiogram, translin-DNA complex migrates at about 36 kDa (lane 2, Fig. 6). No band could be detected in lane 4 loaded with BSA∶DNA irradiated sample, suggesting that crosslinking of DNA with TRAX was not due to artifact A very high molecular mass complex was also detected in both the lanes. Similar high molecular mass band was also detected at much lower concentration in the translin-[α-^32^P]GTP mixture that was irradiated under same conditions.

## Discussion

The nuclease activity of translin-TRAX heteromeric complex has been in recent focus due to its role in siRNA processing [16], [29]. A number of studies also provided evidence that TRAX and translin play important role in DNA damage recovery [12]–[14]. The functional role of these proteins vis-à-vis their DNA-binding ability has also been argued recently [22]. Binding of DNA to the translin-TRAX complex has been thought to be mediated through translin, and TRAX was not expected to bind the nucleic acid substrates [21], [22], [29]. In the present study, we show direct binding of ssDNA with the TRAX protein by crosslinking radiolabeled ssDNA with translin-TRAX complexes using UV-laser. The DNA binding studies were carried out with the heteromeric complexes as TRAX alone is not stable by itself [10], [25], [30]. The heteromeric complexes used in DNA-binding studies were purified by the size exclusion column chromatography and were of molecular masses ∼300 kDa. From the relative intensity of translin or TRAX bands on the SDS-PAGE equimolar ratio of the two proteins in the heteromeric complexes could be taken. Although we expect heteromeric complexes as translin_4_-TRAX_4_ multimers, minor contamination of conformational oligomers of compositions translin_5_-TRAX_3_ or translin_6_-TRAX_2_, given the resolution of Superdex 200 gel filtration column, cannot be ruled out. Post-irradiation, the heteromeric complex was disrupted using chaotropic agent, urea, under boiling condition. The 6×His tagged TRAX/TRAX-DNA crosslinked complex was purified by metal chelating affinity matrix under denaturing conditions, while untagged translin was recovered in extensive wash steps. The TRAX-DNA complex was clearly detected on autoradiograph of SDS-PAGE analysis of the eluent. Also, the translin-DNA covalent complex was detected in the irradiated translin∶DNA mixture. The TRAX-DNA complex migrates corresponding to molecular mass of about 44 kDa, compared to 36 kDa translin-DNA covalent complex (Fig. 6).

Since the UV light is known to induce protein–DNA crosslinking predominantly at their contact points, we tested the hypothesis that the B2 and B3 motifs in TRAX play a critical role in the nucleic acid binding activity of the heteromeric complex. The B2 and B3 motifs of TRAX are juxtaposed to the DNA binding B2/B3 motifs on translin in the recently resolved crystal structures of translin-TRAX complexes [29], [30] (Fig. 2). We mutated residues of these motifs and examined the DNA binding activity of mutant complexes. These mutations did not cause any perturbation in structure or their oligomeric status as wild-type and mutant complexes showed similar CD-spectra and elution profiles on gel-filtration analysis. We found that mutations in the TRAX B3 site drastically reduce the binding activity of the heteromeric complex formed with wild type translin, while mutations in the TRAX B2 site do not. However, the TRAX B2 mutations do impair the ability of TRAX to form fully active heteromeric complexes with the translinB2 mutant. Also, drastic loss of DNA binding activity was observed in heteromeric complex translinB2-TRAXB3 (Fig. 5). Lastly, homomeric or heteromeric complexes with substitutions in the B3 motifs of translin are totally inactive in binding assays (Fig. S2).

The DNA-binding domain of human translin is formed by combination of its basic motifs in a multimeric structure and loss of multimeric structure results in abrogation of its DNA binding abilities [20]. The DNA-binding competent oligomers of translin were earlier shown to be constituted by energetically stable and evolutionarily conserved dimers in up-down configuration [31]. In contrast, TRAX has been shown not to interact with itself using yeast two hybrid system [21]. Additionally, heteromeric complexes have been observed in a variety of TRAX/translin compositions; translin_4_-TRAX_4_ [22], translin_5_-TRAX_3_ and translin_6_-TRAX_2_ [29] and translin_4_-TRAX_2_ [30]. The complexes are constituted by translin-TRAX and translin-translin up-down dimers. Importantly, TRAX-TRAX homodimers are not observed. These observations together hint that TRAX alone cannot oligomerize. It can thus be rationalized that DNA binding activity of TRAX could not be detected in earlier studies since it alone could not achieve nucleic acid binding-competent oligomeric status.

The conformation of translin-TRAX heterodimer closely resembles that of translin-translin dimer in crystal structures [29], [32] (Fig. 2 and Fig. S4). Since mutations in both of the B2 and B3 sites abolish the nucleic acid binding activity of the complex, it can be concluded that B2 and B3 motifs of both translin and TRAX contribute to DNA binding activity. The identified nucleic acid binding motifs reside on the equatorial region of the translin-TRAX octamer. The shortest distance between DNA binding motif and TRAX catalytic centre responsible for RNase activity of translin-TRAX complex is about 9 Å [16], [29]. We anticipate that knowledge of DNA binding domains of TRAX will help guide future studies aimed at elucidating relationship between its high affinity nucleic acid binding and RNase activities of the translin-TRAX complexes.

In summary, we isolated the covalent complex of TRAX with the probe ssDNA after disrupting the UV-irradiated heteromeric translin-TRAX complex. We have identified the B2 and B3 sites in TRAX as mediating its role in nucleic acid binding activity of the heteromeric complex. We suggest that the complex is assembled from translin-TRAX or translin-translin dimer subunits, and B2/B3 motifs of both translin and TRAX primarily contribute to DNA binding activity.

## Supporting Information

Figure S1 **Native-PAGE analysis.** Human translin and its mutants were resolved on 4.5% polyacrylamide gel under native conditions (Lane 1, translin; lane 2, translinB3; lane 4, translinB2). The proteins purified by three-stage chromatography were those eluted at molecular mass of about 236 kDa from Superdex 200 column. The native-PAGE showed presence of low-abundance high molecular mass oligomer.
(TIF)

Figure S2 **DNA-binding activity of the proteins/complexes.** [γ-^32^P]-labeled Bcl-CL1 24-mer ssDNA (200 nM) was incubated with human translin (100 nM of octameric translin) and translin-TRAX complexes (100 nM of translin-TRAX complex). The mixtures were resolved on the 4.5% native-PAGE in TBE buffer. Prior to EMSA analysis all the purified samples were treated with DNase1 and RNaseA overnight at 20°C followed by purification using Ni-IDA chelating sepharose column. Lane 1, human translin; lane 2, human translin-TRAX complex; lanes 3 and 4, complexes of human translin with B2 and B3 mutants of human TRAX, respectively; lane 5, translinB2 mutant; lanes 6, 7 and 8, complexes of translinB2 mutant with wild-type human TRAX, with B2 mutant of human TRAX and with B3 mutant of human TRAX, respectively; lane 9, translinB3 mutant; lanes 10,11 and 12, complexes of translinB3 mutant with wild-type human TRAX, with B2 mutant of human TRAX and with B3 mutant of human TRAX, respectively.
(TIF)

Figure S3 **Supershift analysis of TRAX-DNA complex.** To confirm TRAX-DNA crosslinking, a 43-mer unlabeled DNA was incubated with translin-TRAX complex and was UV-irradiated. Post-irradiation, the complex was disrupted using chaotropic agents and TRAX-DNA complex alone was purified using 6×His tag available on TRAX sequence alone. The covalent complexes of TRAX with 43-mer DNA (lanes 4 and 5), with 24-mer DNA (lanes 1 and 2) and molecular weight markers (lane 3) were resolved on 12% SDS-PAGE. The protein bands were stained with silver-stain. The relative migration of covalent TRAX-DNA complexes (boxed, weak band intensity suggests poor yield of the covalent complex) corresponded to the molecular mass difference between 43- and 24- mer DNA.
(TIF)

Figure S4 **Cartoon showing close proximity of B2 and B3 motifs on up-down dimer of human translin.** The two monomers of translin are shown in cyan and blue colors. The B2 and B3 motifs are shown as spheres. The figure was prepared using atomic coordinates of human translin structure (PDB code,1J1J) and PyMol suite.
(TIF)

Table S1 **Primer sequences used to synthesize the mutants of translin and TRAX.**
(DOCX)
