# Supplementary figures and images for: Identification of Nucleic Acid Binding Sites on Translin-Associated Factor X (TRAX) Protein
Source: PLoS One. 2012 Mar 12;7(3):e33035. doi: 10.1371/journal.pone.0033035 (PMC3299731; doi:10.1371/journal.pone.0033035)

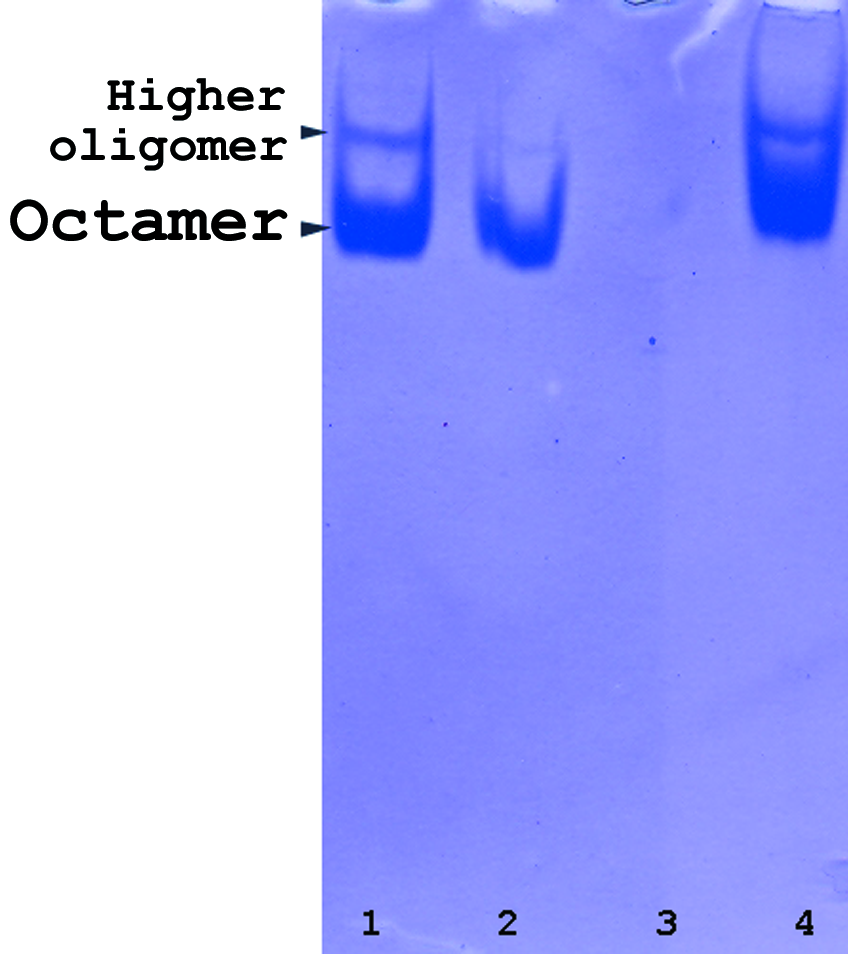

Supplement: Figure S1 — Native-PAGE analysis. Human translin and its mutants were resolved on 4.5% polyacrylamide gel under native conditions (Lane 1, translin; lane 2, translinB3; lane 4, translinB2). The proteins purified by three-stage chromatography were those eluted at molecular mass of about 236 kDa from Superdex 200 column. The native-PAGE showed presence of low-abundance high molecular mass oligomer. (TIF) [file pone.0033035.s001.tif]

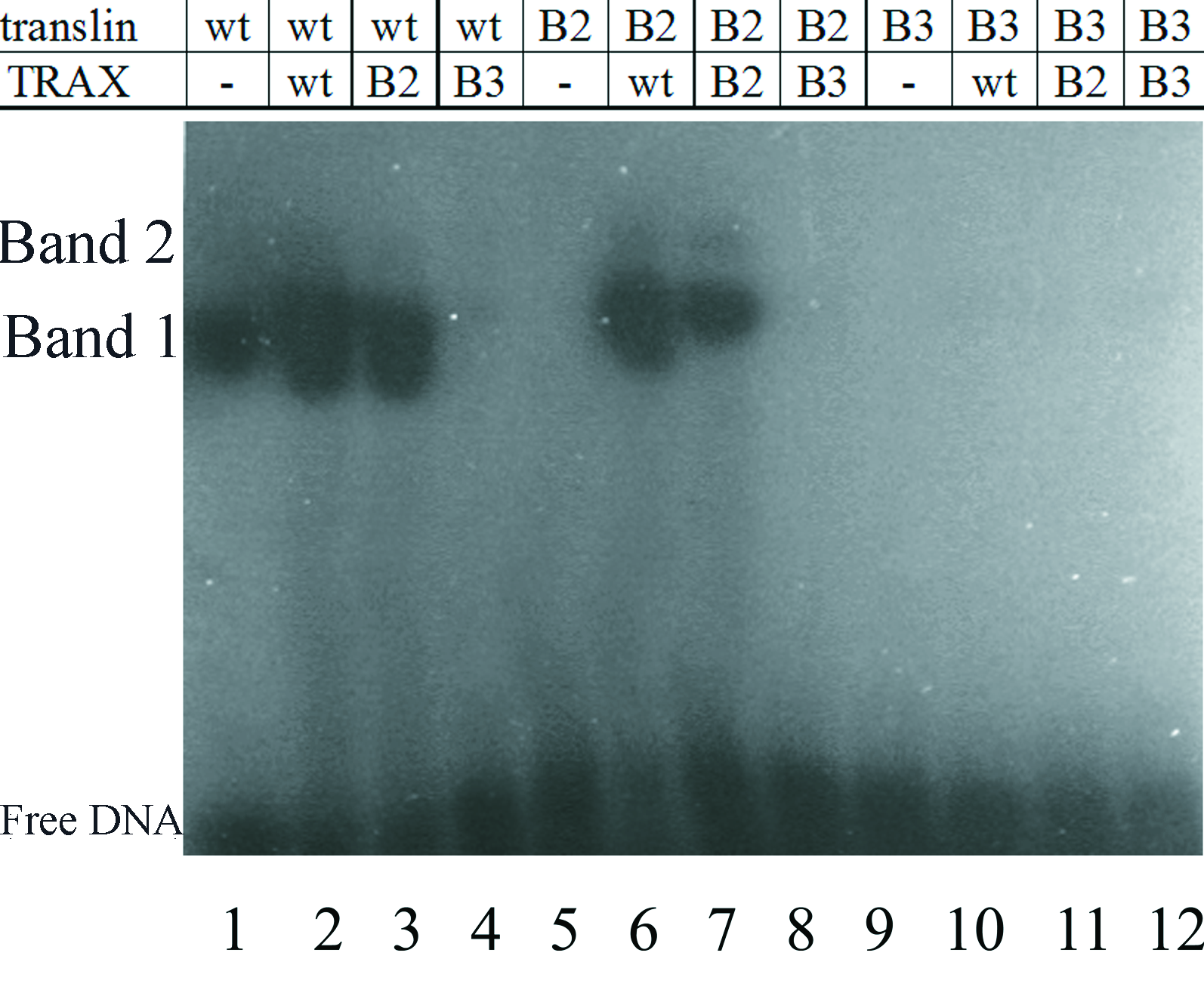

Supplement: Figure S2 — DNA-binding activity of the proteins/complexes. [γ-32P]-labeled Bcl-CL1 24-mer ssDNA (200 nM) was incubated with human translin (100 nM of octameric translin) and translin-TRAX complexes (100 nM of translin-TRAX complex). The mixtures were resolved on the 4.5% native-PAGE in TBE buffer. Prior to EMSA analysis all the purified samples were treated with DNase1 and RNaseA overnight at 20°C followed by purification using Ni-IDA chelating sepharose column. Lane 1, human translin; lane 2, human translin-TRAX complex; lanes 3 and 4, complexes of human translin with B2 and B3 mutants of human TRAX, respectively; lane 5, translinB2 mutant; lanes 6, 7 and 8, complexes of translinB2 mutant with wild-type human TRAX, with B2 mutant of human TRAX and with B3 mutant of human TRAX, respectively; lane 9, translinB3 mutant; lanes 10,11 and 12, complexes of translinB3 mutant with wild-type human TRAX, with B2 mutant of human TRAX and with B3 mutant of human TRAX, respectively. (TIF) [file pone.0033035.s002.tif]

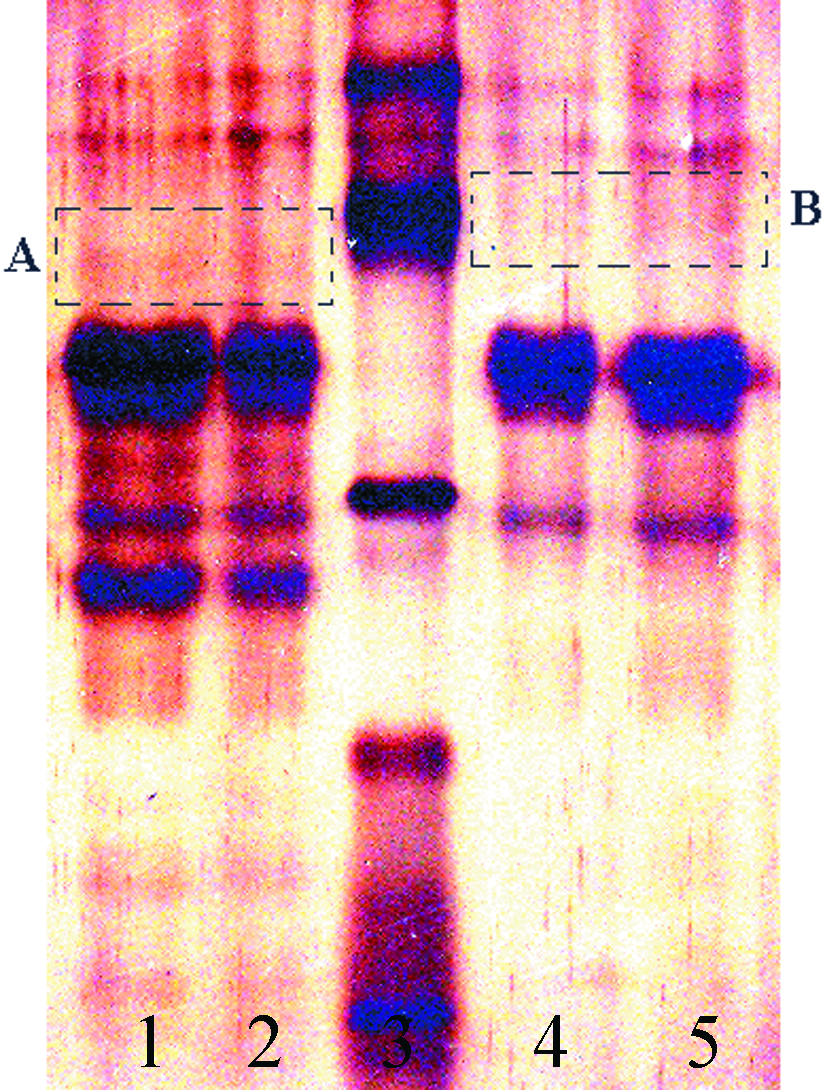

Supplement: Figure S3 — Supershift analysis of TRAX-DNA complex. To confirm TRAX-DNA crosslinking, a 43-mer unlabeled DNA was incubated with translin-TRAX complex and was UV-irradiated. Post-irradiation, the complex was disrupted using chaotropic agents and TRAX-DNA complex alone was purified using 6×His tag available on TRAX sequence alone. The covalent complexes of TRAX with 43-mer DNA (lanes 4 and 5), with 24-mer DNA (lanes 1 and 2) and molecular weight markers (lane 3) were resolved on 12% SDS-PAGE. The protein bands were stained with silver-stain. The relative migration of covalent TRAX-DNA complexes (boxed, weak band intensity suggests poor yield of the covalent complex) corresponded to the molecular mass difference between 43- and 24- mer DNA. (TIF) [file pone.0033035.s003.tif]

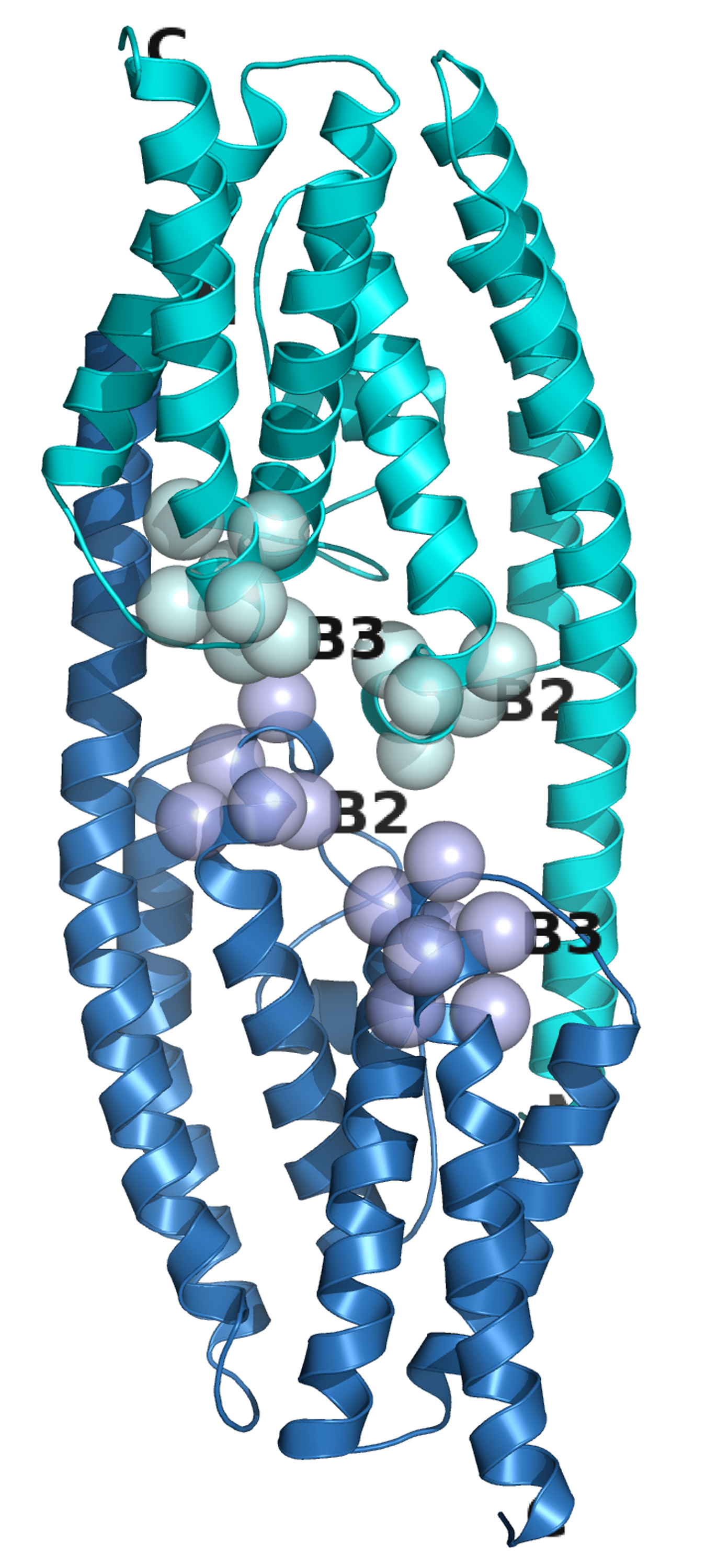

Supplement: Figure S4 — Cartoon showing close proximity of B2 and B3 motifs on up-down dimer of human translin. The two monomers of translin are shown in cyan and blue colors. The B2 and B3 motifs are shown as spheres. The figure was prepared using atomic coordinates of human translin structure (PDB code,1J1J) and PyMol suite. (TIF) [file pone.0033035.s004.tif]
