# Supplementary material for: Identification of Nucleic Acid Binding Sites on Translin-Associated Factor X (TRAX) Protein
Source: PLoS One. 2012 Mar 12;7(3):e33035. doi: 10.1371/journal.pone.0033035 (PMC3299731; doi:10.1371/journal.pone.0033035)
Supplement: Table S1 — Primer sequences used to synthesize the mutants of translin and TRAX. (DOCX) [file pone.0033035.s005.docx]

**Supplementary Materials**

**Materials and Methods**

***Photochemical crosslinking of the heteromeric complex with 43-mer DNA probe***- The heteromeric complex of wild-type human translin and TRAX proteins (50 pmol) of molecular mass about 300 kDa, as purified from the size exclusion chromatography column, was incubated with 50 pmol of the 43-mer unlabeled-DNA oligo (reverse primer used for cloning of *trxB2* mutant, supplementary Table 1) for 30 min in 50 µL binding buffer (25 mM Tris-HCl pH 8.0, 100 mM NaCl, 1 mM EDTA). The protein:DNA mixture was irradiated in a quartz cell (Eppendorf UVette) by UV-laser under the conditions same as used for crosslinking of 24-mer DNA probe. Post-irradiation, TRAX-DNA crosslinked complex was purified after disruption with chaotropic agents following the procedure as used for purification of 24-mer DNA covalent complex. The covalent complexes of TRAX with 24- and 43- mer ssDNA were resolved on 12% SDS-PAGE. The protein bands were stained with silver-stain to analyze supershift of the TRAX-DNA complex due to higher molecular mass of 43-mer DNA. A band (marked with box in supplementary Fig. S3) of poor intensity also suggested very poor yield of protein-DNA crosslinking in UV-irradiation experiments.

**Table 1. Primer sequences used to synthesize the mutants of translin and TRAX.**

| Gene | Forward primer | Reverse primer |
| --- | --- | --- |
| *translinB2* | GCTGAACAGTATTACACATTTAATGAGAACTGGAGGTTTGTGTTG | CAACACAAACCTCCAGTTCTCATTAAATGTGTAATACTGTTCAGC |
| *translinB3* | CCTGAAAAATGACTCCCTGACGAACAGCAACGACGGATTGAAATATGACG | CGTCATATTTCAATCCGTCGTTGCTGTTCGTCAGGGAGTCATTTTTCAGG |
| *trxB2* | CAGGGGAAGATATGCATCTGTTCAATGCAGCCATTACTACAGG | CCTGTAGTAATGGCTGCATTGAACAGATGCATATCTTCCCCTG |
| *trxB3* | CCTTACGAGGTTTCTAATACGCTGAATACCTTGAAACAAAG | CTTTGTTTCAAGGTATTCAGCGTATTAGAAACCTCGTAAGG |

**Figure Legends**

**Figure S1. Native-PAGE analysis.** Human translin and its mutants were resolved on 4.5% polyacrylamide gel under native conditions (Lane 1, translin; lane 2, translinB3; lane 4, translinB2). The proteins purified by three-stage chromatography were those eluted at molecular mass of about 236 kDa from Superdex 200 column. The native-PAGE showed presence of low-abundance high molecular mass oligomer.

**Figure S2. DNA-binding activity of the proteins/complexes**. [γ-^32^P]-labeled Bcl-CL1 24-mer ssDNA (200 nM) was incubated with human translin (100 nM of octameric translin) and translin-TRAX complexes (100 nM of translin-TRAX complex). The mixtures were resolved on the 4.5% native-PAGE in TBE buffer. Prior to EMSA analysis all the purified samples were treated with DNase1 and RNaseA overnight at 20 ^o^C followed by purification using Ni-IDA chelating sepharose column. Lane 1, human translin; lane 2, human translin-TRAX complex; lanes 3 and 4, complexes of human translin with B2 and B3 mutants of human TRAX, respectively; lane 5, translinB2 mutant; lanes 6, 7 and 8, complexes of translinB2 mutant with wild-type human TRAX, with B2 mutant of human TRAX and with B3 mutant of human TRAX, respectively; lane 9, translinB3 mutant; lanes 10,11 and 12, complexes of translinB3 mutant with wild-type human TRAX, with B2 mutant of human TRAX and with B3 mutant of human TRAX, respectively.

**Figure S3**. **Supershift analysis of TRAX-DNA complex**. To confirm TRAX-DNA crosslinking, a 43-mer unlabeled DNA was incubated with translin-TRAX complex and was UV-irradiated. Post-irradiation, the complex was disrupted using chaotropic agents and TRAX-DNA complex alone was purified using 6XHis tag available on TRAX sequence alone. The covalent complexes of TRAX with 43-mer DNA (lanes 4 and 5), with 24-mer DNA (lanes 1 and 2) and molecular weight markers (lane 3) were resolved on 12% SDS-PAGE. The protein bands were stained with silver-stain. The relative migration of covalent TRAX-DNA complexes (boxed, weak band intensity suggests poor yield of the covalent complex) corresponded to the molecular mass difference between 43- and 24- mer DNA.

**Figure S4. Cartoon showing close proximity of B2 and B3 motifs on up-down dimer of human translin**. The two monomers of translin are shown in cyan and blue colors. The B2 and B3 motifs are shown as spheres. The figure was prepared using atomic coordinates of human translin structure (PDB code,1J1J) and PyMol suite.
